# Stress Response and Hearing Loss Differentially Contribute to Dynamic Alterations in Hippocampal Neurogenesis and Microglial Reactivity in Mice Exposed to Acute Noise Exposure

**DOI:** 10.3389/fnins.2021.749925

**Published:** 2021-12-08

**Authors:** Qian Li, Hong Li, Xiuting Yao, Conghui Wang, Haiqing Liu, Dan Xu, Chenxi Yang, Hong Zhuang, Yu Xiao, Rui Liu, Sinuo Shen, Shaoyang Zhou, Chenge Fu, Yifan Wang, Gaojun Teng, Lijie Liu

**Affiliations:** ^1^School of Life Science and Technology, Southeast University, Nanjing, China; ^2^Medical College, Southeast University, Nanjing, China; ^3^School of Public Health, Southeast University, Nanjing, China; ^4^Jiangsu Key Laboratory of Molecular Imaging and Functional Imaging, Department of Radiology, Medical School, Zhongda Hospital, Southeast University, Nanjing, China

**Keywords:** noise exposure, noise-induced hearing loss, stress, hippocampal neurogenesis, microglia

## Abstract

Noise-induced hearing loss (NIHL) is one of the most prevalent forms of acquired hearing loss, and it is associated with aberrant microglial status and reduced hippocampal neurogenesis; however, the nature of these associations is far from being elucidated. Beyond its direct effects on the auditory system, exposure to intense noise has previously been shown to acutely activate the stress response, which has increasingly been linked to both microglial activity and adult hippocampal neurogenesis in recent years. Given the pervasiveness of noise pollution in modern society and the important implications of either microglial activity or hippocampal neurogenesis for cognitive and emotional function, this study was designed to investigate how microglial status and hippocampal neurogenesis change over time following acoustic exposure and to analyze the possible roles of the noise exposure-induced stress response and hearing loss in these changes. To accomplish this, adult male C57BL/6J mice were randomly assigned to either a control or noise exposure (NE) group. Auditory function was assessed by measuring ABR thresholds at 20 days post noise exposure. The time-course profile of serum corticosterone levels, microglial status, and hippocampal neurogenesis during the 28 days following noise exposure were quantified by ELISA or immunofluorescence staining. Our results illustrated a permanent moderate-to-severe degree of hearing loss, an early but transient increase in serum corticosterone levels, and time-dependent dynamic alterations in microglial activation status and hippocampal neurogenesis, which both present an early but transient change and a late but enduring change. These findings provide evidence that both the stress response and hearing loss contribute to the dynamic alterations of microglia and hippocampal neurogenesis following noise exposure; moreover, noise-induced permanent hearing loss rather than noise-induced transient stress is more likely to be responsible for perpetuating the neurodegenerative process associated with many neurological diseases.

## Introduction

In recent decades, a massive shift has been observed in the global burden of disease from communicable causes (e.g., diarrheal and other infectious diseases) to non-communicable causes (e.g., dementia) (Benziger et al., 2016). Dementia is among the most common chronic non-communicable neurodegenerative diseases (Collaborators, 2019; Bazargan-Hejazi et al., 2020). Given the substantial social and economic implications of dementia as well as the lack of curative treatment, an identification and understanding of risk factors associated with dementia are needed to accelerate disease prevention and realize morbidity improvements (Livingston et al., 2017).

Convergent lines of evidence from epidemiological and experimental studies indicate that acquired peripheral hearing loss (HL) is a significant risk factor for dementia (Lin et al., 2011; Liu et al., 2016a; Livingston et al., 2017, 2020; Thomson et al., 2017; Loughrey et al., 2018; Nixon et al., 2019; Orgeta et al., 2019; Uchida et al., 2019). Excessive exposure to environmental noise, a pervasive pollutant that directly affects the health and well-being of exposed subjects (Stansfeld and Matheson, 2003; Uran et al., 2010; Basner et al., 2014; Liu et al., 2016b; Hahad et al., 2019; Zhuang et al., 2020) is the most common contributing factor leading to acquired peripheral HL (Nelson et al., 2005; Jeschke et al., 2020). According to the World Health Organization (WHO), one-third of all cases of HL can be attributed to noise exposure (No authors listed, 1990; Nelson et al., 2005; Le et al., 2017). In our previous study using animals with noise-induced hearing loss (NIHL), impaired cognitive behavior and hippocampal neurogenesis as well as abnormal microglia were observed months after noise exposure (Tao et al., 2015; Liu et al., 2016a,^2018^; Zhuang et al., 2020), which not only provided supportive evidence for the causative role of acquired peripheral HL in the development of dementia but also suggested the mediating roles of hippocampal neurogenesis impairment and microglial dysfunction in this causative relationship. However, beyond its direct effects on the auditory system, exposure to intense noise has previously been shown to acutely activate the stress response (Samson et al., 2007; Liu et al., 2016b; Hahad et al., 2019), which has increasingly been linked to both microglial activity (Frank et al., 2019) and adult hippocampal neurogenesis in recent years (Surget and Belzung, 2021). Given the pervasiveness of noise pollution in modern society (Goines and Hagler, 2007), the potential vulnerability of the hippocampus to intense noise exposure (Kraus et al., 2010; Cui et al., 2012; Cheng et al., 2016; Hayes et al., 2019), and the well-known important implications of either microglial activity or hippocampal neurogenesis for cognitive function (De Lucia et al., 2016; Hansen et al., 2018; Rodriguez-Iglesias et al., 2019; Suss and Schlachetzki, 2020), simultaneously analyzing the time course for changes in the stress response, hearing threshold, microglial status, and hippocampal neurogenesis after cessation of brief intensive noise exposure is essential for better comprehending the deleterious effects of noise on brain function.

Therefore, the main purpose of this study was to investigate how microglial status and hippocampal neurogenesis change over time following acoustic exposure and to analyze the possible roles of noise exposure-induced stress responses and HL in these changes. To accomplish this, adult male C57BL/6J mice were randomly assigned to either a control or noise exposure (NE) group. Auditory function was assessed by measuring auditory brainstem response (ABR) thresholds at 20 days post NE. The dynamic profile of serum corticosterone levels, microglial status, and hippocampal neurogenesis over the time span from immediately after to 28 days after the cessation of NE were analyzed by ELISA or immunofluorescence staining. Our results illustrated a permanent moderate-to-severe degree of NIHL, an early but transient increase in serum corticosterone levels, and dynamic alterations in microglial activation status and hippocampal neurogenesis, which both consist of an early but transient change and a late but enduring change. These findings corroborate previous observations demonstrating the association between NIHL and hippocampal neurodegenerative changes (Liu et al., 2016a,^2018^; Zhuang et al., 2020) and provide further insights into the potential mechanisms underlying the adverse effect of NE on the brain.

## Materials and Methods

### Animals

Young adult (aged 2 months) male C57BL/6J mice were purchased from SPF Biotechnology Co., Ltd. (Beijing, China; No. SCXK 2019-0010). In total, 64 mice that passed the Preyer reflex test were used in the study. The mice were housed four per cage under standard conditions (8 a.m.–8 p.m. light cycle, 22°C, 55% humidity, and *ad libitum* access to food and water) provided by the University Committee for Laboratory Animals of Southeast University, China (SCXK2011-0003). One week after arrival, the mice were randomly assigned to a control group and seven NE groups (*n* = 8/group). Animals in each NE group were separately exposed to broadband noise for 2 h at a sound pressure level (SPL) of 123 dB. The subgroups of NE animals were separately sacrificed immediately after or at 12 h, 1, 3, 7, 14, and 28 days after NE (0HPN, 12HPN, 1DPN, 3DPN, 7DPN, 14DPN, and 28DPN, respectively).

### Noise Exposure

The animals in the NE groups were exposed to a single dose of broadband noise at 123 dB SPL for 2 h during the light phase as previously described (Liu et al., 2016a,^2018^; Zhuang et al., 2020). The awake and unrestrained animals were kept individually in a metal wire mesh cage (cage size: 11 cm × 11 cm × 23 cm) located 40 cm below the horns of two loudspeakers (an NJ speaker YD380-8bH and a BM professional speaker HG10044XT, nominal bandwidth 1–20 kHz). The noise signal was generated by a System III processor from Tucker–Davis Technologies (TDT, Alachua, FL, United States) and amplified by a Yamaha P9500S power amplifier. Animals were acclimatized for 30 min in the exposure chamber before the loudspeakers were turned on. During the exposure, which began at 06:00 p.m., the noise level was monitored and controlled within the range of 123 dB SPL ± 1 dB by using a 1/4-inch microphone linked to a sound level meter (Larson Davis, Depew, NY, United States; 2,520 microphone and 824 sound level meter, Larson Davis, Depew, NY, United States). For the control group, all operations were the same as those in the 0 HPN group except that the animals were treated with sham exposure, in which the loudspeaker was not turned on.

### Auditory Brainstem Response Assessment

A number of studies in animal models have demonstrated that, after NEs that are intense enough to produce permanent effects, hearing thresholds recover exponentially for periods extending up to 2∼3 weeks and finally reach a steady state (Kujawa and Liberman, 2006; Ryan et al., 2016). Thus, the ABRs of 28DPN and control mice were assessed 8 days before their sacrifice (i.e., 20 days after NE) in a sound-attenuating chamber to determine the hearing thresholds of mice. The animals were anesthetized by intraperitoneal injection of pentobarbital (50 mg/kg body weight), and their body temperature was maintained at approximately 37.5 ± 0.5°C by placing them on a thermostatic heating pad during testing and recovery from anesthesia. Three subdermal needles were inserted ventrolateral to the left pinna (active), vertex (reference), and right hind limb (ground) of the testing mice, and they served as electrodes for ABR recording. TDT hardware (RZ6, System III) and software (BioSig and SigGen) were used for signal generation and ABR acquisition. Tone bursts of 2, 4, 8, 16, and 32 kHz (10-ms duration with cos^2^ gating and 0.5-ms rise/fall time, at the rate of 21.1/s) were presented monaurally in an open field using a broadband speaker (MF1; TDT) located 10 cm in front of the animal’s head. The evoked responses were amplified 20 times *via* an RA16PA preamplifier (TDT) and filtered between 100 and 3,000Hz. The responses were averaged 1,000 times. At each frequency, the test was performed in a degressive sequence starting from 90 dB SPL, which was weakened in 5-dB steps until no ABR response was detected. The ABR threshold at each frequency was defined as the lowest sound level at which a repeatable wave III in ABR was detected. If a repeatable wave III was not detected at an SPL of 90 dB, then a threshold of 95dB was assigned to the mouse.

### Tissue and Blood Harvest

Sample collections of each group except the 12 HPN group were performed between 08:00 and 09:00 a.m. to avoid potential variations related to the circadian rhythm; for the 12 HPN group, the collections were performed between 08:00 p.m. and 09:00 p.m. At the defined time of sacrifice, mice were deeply anesthetized with pentobarbital (100 mg/kg, i.p.) and blood was rapidly collected by cardiac puncture into centrifugal tubes and allowed to clot. After blood collection, the animals were perfused transcardially with 20 ml 0.9% saline followed by 20 ml of 4% paraformaldehyde (PFA) in 0.1 M PBS, and whole brains were quickly excised, postfixed in 4% PFA for 6–8 h at 4°C, cryoprotected in 30% sucrose in PBS until the organ sank, embedded in OCT compound, and then stored at −80°C until ready for sectioning.

### Measurement of Corticosterone Levels

The clotted blood samples were centrifuged to separate the cells from the serum. The resulting serum was collected and stored at –80°C until use. The amount of corticosterone in serum was analyzed by enzyme-linked immunosorbent assay (ELISA) using a commercial mouse corticosterone quantification kit (CSB-E07969 m; Cusabio, Houston, TX 77054, United States).

### Immunohistochemistry

Hippocampal neurogenesis and the microglia status in each region of interest (ROI) were determined by immunohistochemistry as previously described (Zhuang et al., 2020). The frozen brain blocks were cut into 40-μm-thick sections using a cryostat (Leica Cryostat Microtome 1900, Heidelberger, Germany). All sections were collected and stored in cryoprotectant solution (30% ethylene glycol and 25% glycerin in 0.1 M phosphate buffer) at –20°C until needed. For each animal, five to six sections (320 μm apart) across the hippocampus were included for the study of neurogenesis or microglia status. For the ventral cochlear nucleus (VCN), dorsal cochlear nucleus (DCN), inferior colliculus (IC), and auditory cortex (AC), two to three coronal sections per ROI (sections 120 μm apart for the VCN and DCN and 320 μm apart for the IC and AC) per animal were used for microglial analysis.

Staining was performed using selected free-floating sections. The selected sections were permeabilized with 0.1% Triton X-100 in PBS for 30 min. After blocking with blocking solution for 2 h, the following primary antibodies were incubated overnight at 4°C: rabbit anti-Ki67 (for proliferating cells; Abcam, 1:500, ab16667), guinea pig anti-DCX (doublecortin, for newly generated neurons; Millipore, 1:1,000, AB2253), rabbit anti-Iba1 (ionized calcium-binding adaptor molecule 1, for microglia; Wako, 1:1,500, 019-19741), and rat anti-CD68 (cluster of differentiation 68, for phagocytic microglia; Bio-Rad, 1:1,500, MCA1957). After extensive washing, the sections were incubated with the appropriate secondary antibody for 2 h at room temperature: Alexa-488 goat anti-rabbit (Abcam, Cambridge, CB2 0AX, United Kingdom, 1:1,000, ab150077), Alexa-568 goat anti-guinea pig (Abcam, 1:1,000, ab175714), Alexa-568 goat anti-rabbit (Abcam, 1:1,000, ab175471), and Alexa-488 goat anti-rat (Thermo Fisher Scientific, 1:1,000, A11006). All sections were counterstained with DAPI (Beyotime, Shanghai, China, 1:750, C1027) at room temperature for 15 min to visualize the cell nuclei.

### Image Analysis

Images were captured using a fluorescence microscope (Olympus BX53, Japan) or a confocal microscope (Olympus FV3000, Japan). Samples were analyzed by an observer blinded to the experimental treatment using ImageJ software (US National Institutes of Health, Bethesda, MD, United States). Maximum intensity projections of confocal z-series stacks at an interval of 1.0 μm were created and aligned in the x–y plane to create two-dimensional images. The target brain regions of interest (ROIs) were confirmed according to the Allen Mouse Brain Atlas (Allen Institute for Brain Science, 2008). The number of DCX- or Ki67-positive cells in each image was manually counted using the cell counter function of ImageJ in an area of the subgranular zone (SGZ), which is defined as the SGZ length in the image multiplied by an SGZ height of 20 μm [a layer of cells expanding 5 μm into the hilus and 15 μm into the granular cell layer (GCL)] (Sierra et al., 2010; Zhuang et al., 2020).

Microglial cells were identified by immunofluorescence staining using Iba1-specific antibodies, enabling an analysis of the morphology of individual microglial cells (Kreisel et al., 2014; Young and Morrison, 2018). The following parameters were employed to characterize the microglial phenotypic profile (Gebara et al., 2015; Hong et al., 2016; Fernandez-Mendivil et al., 2021) in the defined brain ROIs: (1) microglial density, which was defined as the number of Iba1-positive (Iba1^+^) cells per brain area; (2) average microglial soma area, which was defined as the area of the spherical part of Iba1^+^ cells that contains the nucleus; (3) average microglial territory area, which was defined as the two-dimensional area formed by connecting the outermost points of an Iba1^+^ cell’s dendritic processes; and (4) microglial CD68 score, which was determined by the CD68 occupancy within Iba1^+^ microglia, ranging from 0 (low CD68 occupancy) to 3 (high CD68 occupancy), where a higher score corresponded to higher microglial phagocytic activity.

### Statistical Analysis

The data were analyzed using GraphPad Prism 5 (GraphPad Software). All values are expressed as the mean ± standard error (SE). The level of statistical significance between groups was determined using one- or two-way analysis of variance (ANOVA) followed by Tukey’s or Dunnett’s *post hoc* test or Student’s two-tailed *t*-test as appropriate. Relationships between parameters were assessed by Pearson’s coefficient analysis. Values of *p* < 0.05 were accepted as statistically significant.

## Results

### Permanent Hearing Loss Induced by Noise Exposure

**FIGURE 1:**
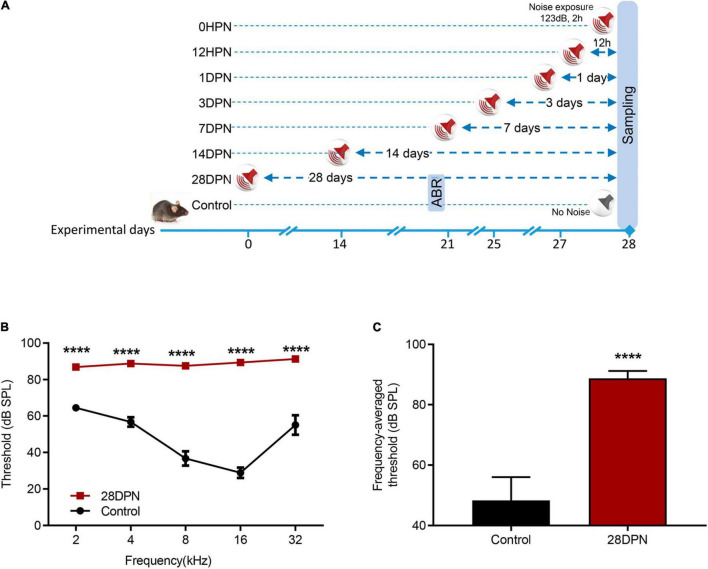
Noise-induced ABR threshold shift measured at 20 days post noise exposure. **(A)** Experimental design for noise exposure and sampling. **(B)** ABR frequency-threshold curves of the 28DPN and control groups obtained at 20 days after noise exposure. **(C)** ABR frequency-averaged thresholds for the 28DPN and control groups obtained at 20 days after noise exposure. The values are presented as the mean ± SEM of eight mice per group. ****p < 0.0001 in the *post hoc* comparisons between the 28DPN group and the control group using two-way repeated-measure ANOVA **(B)** or *t*-test **(C)**.

Because auditory thresholds caused by NE recover exponentially with increasing post exposure time and reach a steady state within 2∼3 weeks (Kujawa and Liberman, 2006; Ryan et al., 2016), ABR audiograms of the 28DPN group were obtained at 20 days post exposure and compared with the time-matched value from the control group to evaluate the extent of permanent NIHL (Figure 1A). Figure 1B shows that the ABR threshold was significantly higher in 28DPN mice than in control mice at every tested frequency [two-way ANOVA, effect of noise: *F*_(1_, _75)_ = 498.5, *p* < 0.0001]. The frequency-averaged threshold (Figure 1C) of the 28DPN group was significantly higher than that of the control group (88.75 ± 0.8814 dB SPL vs. 48.33 ± 2.571 dB SPL, *p* < 0.0001). These results indicated that a moderate-to-severe degree of permanent hearing loss was induced by the NE experiments.

### Early but Transient Stress Response Induced by Noise Exposure

**FIGURE 2:**
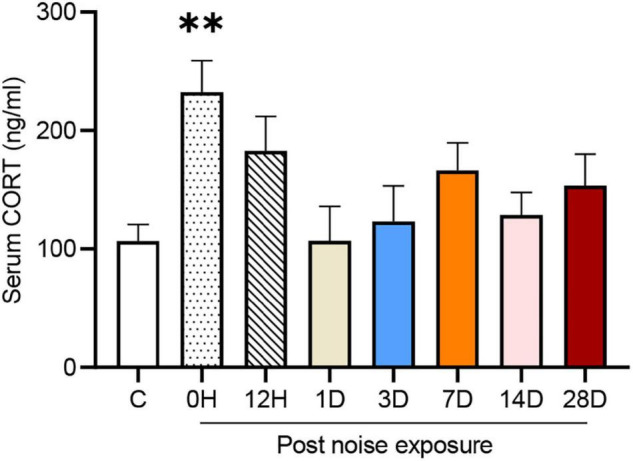
Noise-exposed mice exhibited an early but transient increase in serum corticosterone (CORT). The values are presented as the mean ± SEM of 6–8 mice per group. ^**^p < 0.01 in the *post hoc* comparisons between the noise group and the controls after one-way ANOVA.

The serum corticosterone (CORT) concentration of each group is shown in Figure 2. One-way ANOVA followed by Dunnett’s multiple-comparison test revealed that only the 0 HPN group exhibited significantly elevated serum CORT levels compared with the control group (*p* = 0.0044), indicating that an immediate but transient stress response was induced by the NE experiments.

### Dynamic Changes in Hippocampal Neurogenesis After Noise Exposure

To evaluate the impact of acute high-intensity NE on hippocampal neurogenesis, we stained the hippocampal sections for Ki67 (an endogenous proliferative marker of adult neurogenesis) and DCX (an endogenous marker of newly generated neurons expressed for a duration of 20–30 days after cell division) and counted the number of immunopositive cells.

**FIGURE 3:**
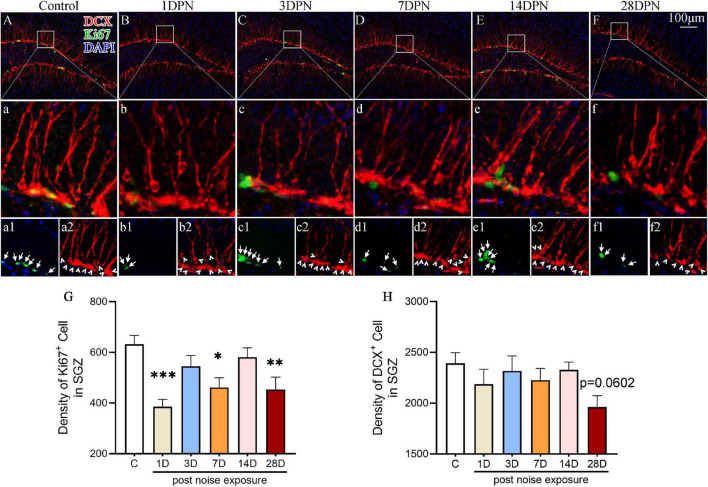
Noise-exposed mice exhibited dynamic changes in hippocampal neurogenesis. **(A–F)** Representative images of DCX^+^ (red) and Ki67^+^ (green) cells in the DG of control and noise-exposed mice. Scale bar: 100 μm. The white rectangle shows the field of view that is shown in the row **(a–f)** below in higher magnification and the third row **(a1–f2)** for Ki67^+^ cells (green, indicated by arrows in **a1–f1**) and DCX^+^ cells (red, indicated by arrowheads in **a2–f2**). **(G,H)** Quantitative analyses of Ki67^+^ cells **(G)** and DCX^+^ cells **(H)** in the SGZ of each group. The values are presented as the mean ± SEM of 8 mice per group. *p < 0.05, ^**^p < 0.01, ^***^p < 0.001 in *post hoc* comparisons between the noise-exposed group and the control group following one-way ANOVA.

Figures 3A–F shows representative images of DCX and Ki67 immunostaining in the SGZ from each group. One-way ANOVA followed by Dunnett’s multiple-comparison test revealed that the noise-exposed mice exhibited significantly fewer Ki67 + cells than the control mice at 1DPN, 7DPN, and 28DPN (Figure 3G) and clearly fewer DCX^+^ cells than the control mice at 28DPN (Figure 3H, *p* = 0.0602).

### Dynamic Alterations of Microglia in Auditory Brain Regions After Acute Noise Exposure

**FIGURE 4:**
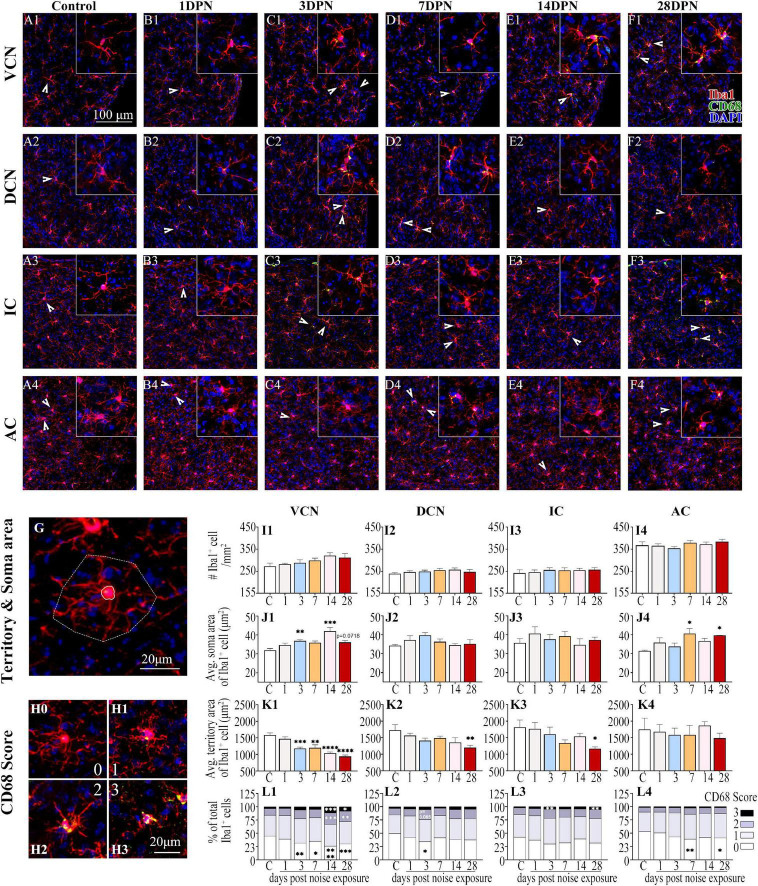
Noise-exposed mice exhibited dynamic microglial alterations in auditory brain regions. **(A1–F4)** Representative images of Iba1 (red)-, CD68 (green)- and DAPI (blue)-stained brain sections of the control and noise-exposed mice. Scale bar: 100 μm. The insets show higher magnifications of the corresponding microglia signified by arrowheads. **(G)** Soma area and territory area of microglia delineated by a solid (soma area) line and a dotted (territory area) line, respectively. **(H0–H3)** Representative images of the scoring of CD68 levels within Iba1^+^ cells. A score of 0 indicates no/scarce expression **(H0)**; 1 signifies only patchy positivity **(H1)**; 2 represents punctate expression roughly covering one-third to two-thirds of cells **(H2)**; and 3 represents greater than two-thirds occupancy **(H3)**. **(I1–L4)** Quantification of the impact of NE on each individual parameter of microglia in the VCN, DCN, IC, and AC. The values are presented as the mean ± SEM of 3–6 mice per group. *p < 0.05, ^**^p < 0.01, ^***^p < 0.001, ^****^p < 0.0001 in *post hoc* comparisons between the noise-exposed group and the control group following one-way ANOVA.

Figures 4A1–F4 show representative confocal images of microglial cells stained for Iba-1 and CD68 from each group across auditory brain ROIs. Similar to previous observations (Olah et al., 2011; Zhuang et al., 2020), microglia in the control mice exhibited ramified morphologies that differed in cell density and process ramification at distinct anatomical regions of the brain (Figures 4A1–4). Compared with the control group, the noise-exposed groups showed comparable microglial density in each ROI. Microglia with larger somas, fewer branches, thicker processes, and increased CD68 occupancy were more frequently observed in noise-exposed groups, indicating microglial activation caused by NE (Hong et al., 2016; Zhuang et al., 2020).

Consistent with previous studies (Campos Torres et al., 1999; Baizer et al., 2015; Zhuang et al., 2020), quantification of the morphological features of microglia (Figures 4G,I1–L4) revealed that the microglia in the VCN, DCN, IC, and AC assumed an activated phenotype at 28DPN characterized by increased soma area (Figures 4J1,J4), decreased microglial territory area (Figures 4K1–3), increased percentage of microglia with high CD68 score, but decreased percentage of microglia with lower CD68 score (Figures 4L1–4). Microglia in the VCN were persistently activated from 3DPN to 28DPN (Figures 4J1,K1,L1). Although few signs of microglial activation were observed in the DCN and IC at 3DPN (Figures 4L2,L3) and the AC at 7DPN (Figures 4J4,L4), no signs of microglial activation were observed in the auditory ROIs at 14DPN except for the VCN.

### Dynamic Alterations in Hippocampal Microglia After Acute Noise Exposure

**FIGURE 5:**
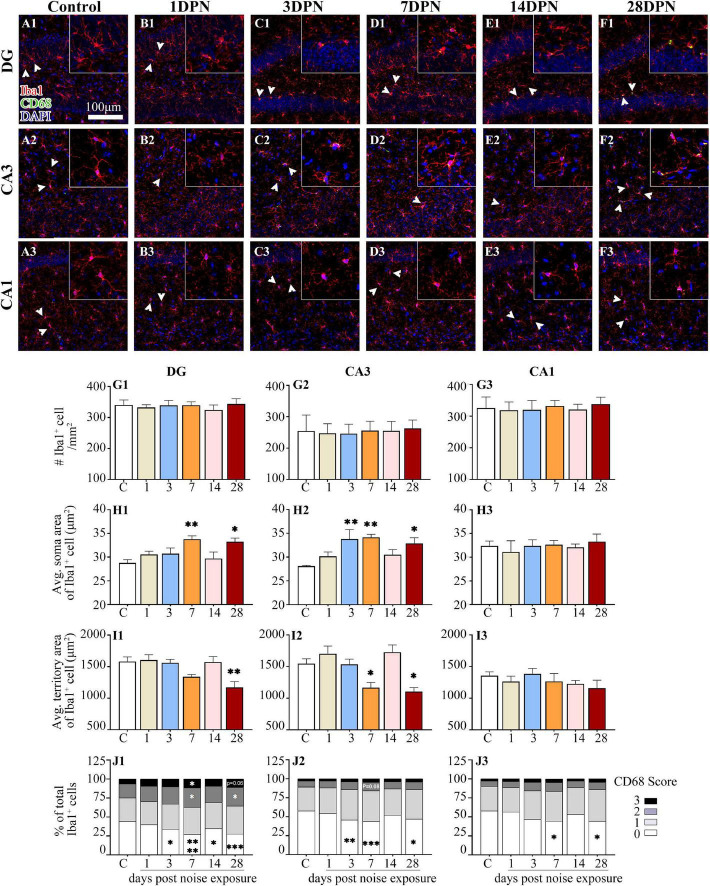
Noise-exposed mice exhibited dynamic microglial alterations in the hippocampus. **(A1–F3)** Representative images of Iba1 (red)-, CD68 (green)-, and DAPI (blue)-stained brain sections of control and noise-exposed mice. Scale bar: 100 μm. The insets show higher magnifications of the corresponding microglia signified by arrowheads. **(G1–J3)** Quantification of the impact of NE on each individual parameter of microglia in the DG, CA3, and CA1. The values are presented as the mean ± SEM of 3–6 mice per group. *p < 0.05, ^**^p < 0.01, ^***^p < 0.001, ^****^p < 0.0001 in *post hoc* comparisons between the noise-exposed group and the control group following one-way ANOVA.

As shown in Figure 5, Iba1^+^ microglial cells were widely distributed in the DG, CA3, and CA1 of both the control and noise-exposed mice. Similar to our previous study (Zhuang et al., 2020), microglia in the control mice exhibited ramified morphologies that differed in cell density and process ramification at distinct anatomical regions of the hippocampus (Figures 5A1–3). Microglial density in the ROIs was comparable between the groups. However, microglia with enlarged soma, decreased territory area, and increased CD68 occupancy were more frequently observed in the noise-exposed mice (Figures 5B1–F3), suggesting that an abnormal hippocampal microglial reaction developed in the noise-exposed mice.

The time-course profile of hippocampal microglial parameters following NE is shown in Figures 5G1–J3. *Post hoc* comparisons using Dunnett’s test indicated microglial overactivation in the DG and CA3 at 28DPN, as evidenced by a significant increase in the soma area (Figures 5H1,H2), decrease in the territory area (Figures 5I1,I2), increase in the percentage of microglia with higher CD68 score (Figure 5J1), and/or decrease in the percentage of microglia with lower CD68 score (Figures 5J1,J2). Signs of microglial activation (i.e., significantly larger than the control soma area, smaller than the control territory area, and enhanced CD68 expression reflected by the CD68 score) were observed in the DG and CA3 at 3DPN and 7DPN but disappeared at 14DPN. Except for the decreased percentage of microglia with lower CD68 scores exhibited at 7DPN and 28DPN (Figure 5J3), no sign of abnormal microglial status was observed in CA1.

## Discussion

Acquired peripheral HL has been ranked as the largest potentially modifiable health and lifestyle factor for the development of dementia (Lin et al., 2011; Livingston et al., 2017, 2020; Thomson et al., 2017; Nixon et al., 2019; Orgeta et al., 2019; Uchida et al., 2019). Environmental noise represents the most common preventable cause of acquired sensorineural hearing loss, and it is increasingly encountered in many aspects of day-to-day modern life (Rabinowitz, 2000; Le et al., 2017). Worldwide, 16% of the disabling hearing loss in adults is attributed to occupational noise including construction, agriculture, mining, manufacturing, utilities, transportation, and the military (Chepesiuk, 2005; Nelson et al., 2005). NIHL resulting from hazardous recreational noise (e.g., sporting events, concerts, nightclubs, firearms, and personal stereos) is increasing in modern society (Meinke et al., 2017; Feder et al., 2019; Gopal et al., 2019). Popular “boom cars” equipped with powerful stereo systems that are usually played with the volume and bass turned up abnormally high, and the car windows rolled down can hit 140–150 dBA (Chepesiuk, 2005). It is estimated that 12% or more of the global population is at risk of hearing loss due to unsafe levels of sound exposure (Le et al., 2017). According to WHO reports, one-third of all cases of hearing loss can be attributed to NE No authors listed (1990), and the prevalence of NIHL is still increasing (Imam and Hannan, 2017). Considering the tremendous disability weight and the expanding healthcare burden posed by dementia (Livingston et al., 2020) as well as the increasing pervasiveness of noise (Goines and Hagler, 2007), the animal model of NIHL is undoubtedly optimal for studying the underlying mechanisms linking dementia and acquired peripheral HL.

The hippocampus, a kernel cognition- and emotion-related brain region embedded deep into the temporal lobe, is generally considered one of the earliest affected brain regions in patients with dementia (Braak and Braak, 1997; Xue et al., 2019). Adult hippocampal neurogenesis is a unique form of neural circuit plasticity that results in the generation of new neurons in the dentate gyrus (DG) throughout life and plays a vital role in hippocampus-dependent learning and memory (Sahay et al., 2011; Lazarov and Hollands, 2016). Adult hippocampal neurogenesis generates only granule cells, the principal neuron in the DG (Kempermann et al., 2015). The first relay in the hippocampal trisynaptic circuit is composed of granule cells, which extend dendrites into the molecular layer and present axons that form the mossy fiber tract that extends to CA3, and these cells are thought to be essential to cognitive function (GoodSmith et al., 2017). Emerging evidence has indicated that altered hippocampal neurogenesis represents an early critical event in the course of dementia (Mu and Gage, 2011; Tobin et al., 2019; Disouky and Lazarov, 2021).

Adult neurogenesis is a complex multistage and multiweek process involving cell proliferation, neuronal differentiation, and, ultimately, survival, and integration into functional circuits (Lagace et al., 2010; Kempermann et al., 2015). This dynamic process is regulated both positively and negatively by a variety of growth factors and environmental experiences (Zhao et al., 2008). Microglia, the resident macrophages and primary immune cells of the brain, can orchestrate their highly plastic, context-specific adaptive responses to remodel neuronal circuit structures and functions (Wake et al., 2013; Delpech et al., 2015). They play integral roles in both the healthy and injured brain, from surveillance and monitoring to sculpting neuronal circuits and guiding plasticity (Sierra et al., 2010; Gemma and Bachstetter, 2013; Sato, 2015; Chugh and Ekdahl, 2016). Increasing evidence indicates that microglial cells indeed exert vital roles in the maintenance of the functional neurogenic niche and are actively involved in crucial steps of adult neurogenesis, including the proliferation, differentiation, and survival of newborn cells (Gemma and Bachstetter, 2013; Kohman and Rhodes, 2013; Sato, 2015; Mosser et al., 2017).

Microglial cells are heterogeneous and dynamically pleomorphic. It is well established that the morphology of microglia is inextricably linked to their functional status (Ajami et al., 2007; Angelova and Brown, 2019). Surveillance microglia display a ramified phenotype marked by a stable number, small soma, and numerous long, thin, motile processes with delicate arborization that constantly monitor their immediate surroundings by extending and retracting their processes (also called ramified microglia) (Davalos et al., 2005). If signs of damage are detected, the microglia convert to an activated or reactive state and assume an amoeboid-like phenotype, characterized by shorter, thicker processes and a larger soma, with increased expression of phagocytic markers, such as CD68 (Nimmerjahn et al., 2005; Olah et al., 2011; Tejera and Heneka, 2016; Angelova and Brown, 2019). Many neurodegenerative diseases, such as Parkinson’s disease and Alzheimer’s disease, are associated with abnormal functional phenotypes of microglia (Sarlus and Heneka, 2017; Subramaniam and Federoff, 2017; Baik et al., 2019).

In our previous studies (Liu et al., 2016a,^2018^; Zhuang et al., 2020), a single noise (identical to the present noise setting) was used to create permanent HL in CBA mice, a mouse strain that has been demonstrated to maintain good hearing threshold sensitivity well into old age (Spongr et al., 1997; Kujawa and Liberman, 2006). Cognitive function was evaluated by the Morris water maze (MWM) task at 3 months post exposure (Liu et al., 2016a,^2018^), and hippocampal neurogenesis and microglial status were monitored for up to 12 months following NE by quantitative immunohistochemical analysis of Ki67 (a proliferating cell marker), doublecortin (DCX; an immature progenitor cell marker), Iba1 (a microglia marker), and CD68 (a lysosomal marker indicative of phagocytic activity of microglia) (Liu et al., 2016a,^2018^; Zhuang et al., 2020). We observed that CBA mice with NIHL exhibited prolonged significant cognitive impairment accompanied by marked hippocampal neurogenesis decline (Liu et al., 2016a,^2018^). Our observations not only provide compelling evidence for the causal role of HL in the development of cognitive impairment (Liu et al., 2016a,^2018^) but also suggest that the accelerated age-related hippocampal neurogenesis decline and persistent microglial dysfunction may contribute to the cognitive deficiency that occurs in animals with NIHL (Zhuang et al., 2020). However, the exact mechanisms leading to alterations in hippocampal neurogenesis and microglial status in NIHL mice remain largely mysterious.

Microglia are versatile modulators of neurogenesis, and their influence is dependent on their activation status. Recent findings in human and animal studies indicated that microglial activation inversely correlates with hippocampal volume in neurodegenerative diseases with dementia, providing compelling evidence for the central role of microglial activation in neurodegenerative diseases (Femminella et al., 2016; Salter and Stevens, 2017; Suss and Schlachetzki, 2020). Consistent with our previous observation in CBA mice (Zhuang et al., 2020), C57BL/6J mice subjected to brief NE at high intensity developed permanent hearing loss and exhibited decreased hippocampal neurogenesis and aberrant microglial activation at 28DPN, thus providing further support for the assumption that hippocampal microglial dysfunction might contribute to acquired hearing loss-related hippocampal neurogenesis (Zhuang et al., 2020).

The brain is a finely tuned machine that has a fascinating capacity to continually undergo structural and functional changes in response to environmental inputs and organism needs; that is, it exhibits neural plasticity (Kleim and Jones, 2008; Sale et al., 2014). Neural circuits that are frequently used have strong connections, while those not actively engaged in task performance for an extended period of time begin to degrade, i.e., they present a “use-it-or-lose-it” characteristic (Kleim and Jones, 2008). Microglia play a fundamental role in activity-dependent neuroplasticity. It is well established that microglia can quickly adapt to their environment and modify their functions with a broad spectrum of activation states (Li and Barres, 2018). For instance, these cells can respond to light deprivation and reexposure by changing their morphology and modulating their interactions with neuronal circuits, notably regulating processes that include adult hippocampal neurogenesis and actively contributing to the experience-dependent modification or elimination of a specific subset of synapses in the brain (Tremblay et al., 2010).

In line with previous reports on long-term microglial activation in the cochlear nucleus following inner ear damage that reduced neural output from the cochlea (Campos Torres et al., 1999; Baizer et al., 2015), the present study demonstrated microglial activation in the auditory ROIs of NIHL mice at 28 days after the cessation of NE. Considering the “use-it-or-lose-it” principle of neuroplasticity (Syka, 2002; Kleim and Jones, 2008) and the microglial contribution to neuroplasticity (Wake et al., 2013; Valero et al., 2016), it is plausible to assume that microglial activation represents auditory circuit remodeling following acquired hearing loss (auditory deprivation).

Consistent with previously published data from laboratory experiments (Zhuang et al., 2020), the present study demonstrated a significant effect of NE on the functional phenotypes of microglia in the hippocampal DG and CA3 region but not in the CA1 region. The discrepancy in the outcome after NE between subregions could at least be partially explained by a report indicating that the DG-CA3 subregion is extremely sensitive to changes in information input and plays a key role in auditory information processing while the CA1 subregion is highly resistant to changes in sensory input (Holden et al., 2012). Mounting evidence demonstrates that microglia, the well-known key facilitators of neuronal plasticity, can sense and respond to neuronal activity in a variety of contexts (Umpierre and Wu, 2021). The microglial status is subtly altered by local neuronal activity (Ronzano et al., 2021). As the hippocampus is generally believed to be involved in auditory perception (Moxon et al., 1999; Kraus and Canlon, 2012), it is reasonable to hypothesize that abnormal local neuronal activity caused by a disturbance of auditory input is a major contributor to hippocampal microglial overactivity in mice with acquired peripheral HL.

Microglia are well-known major sources of proinflammatory cytokines and the principal target cells of cytokines (Hanisch, 2002). Chronically activated microglia secrete excessive pro-inflammatory cytokines, which can further induce microglial responses toward a dysregulated phenotype (Voet et al., 2019). Microglial-derived cytokine signals are assumed to propagate through the brain by volume transmission (i.e., diffuse through cerebral spinal fluid, nerve bundles, and perivascular space) and wiring (i.e., through neuronal projections and gap junctions) (Vitkovic et al., 2000; Almeida-Suhett et al., 2017). The hippocampus and the auditory regions make reciprocal connections with each other (Kraus and Canlon, 2012), which would enable the diffusion of cytokines produced by chronically activated microglia in the auditory region to the hippocampus through wiring and/or cerebral spinal fluid. Thus, it cannot be excluded that chronically activated microglia in the auditory region might represent one contributor to the microglial status transition in the hippocampus. However, this hypothesis remains highly speculative in the absence of convincing evidence. Hearing loss (threshold sensitivity loss) from extensive loud noise may occur immediately. The deafening effect of NE includes reversible and irreversible components. After exposure that is intense enough to produce permanent effects, hearing thresholds recover exponentially with increasing post exposure time and reach a steady state within 2∼3 weeks (Kujawa and Liberman, 2006). Thus, the significant threshold shift measured at 20 days post exposure represents the magnitude of permanent hearing loss induced by NE in the present study. Moreover, it undoubtedly implies an even greater continuous hearing loss across the 20 days post noise. Intriguingly, although changes in microglia similar to those observed at 28DPN were observed as early as 3DPN, almost no signs of microglial abnormalities were observed at 14DPN in ROIs except the VCN. A similar near-perfect correspondence was seen in hippocampal neurogenesis. These observations provide further evidence for the causal role of permanent HL in microglial dysfunction and hippocampal neurogenesis decline observed long after NE (Zhuang et al., 2020), and they suggest that factors other than HL might serve as a major contributor to the overall alterations that occurred earlier after NE.

Beyond the well-known direct deleterious effects on the auditory system (i.e., hearing loss), exposure to intense noise has previously been shown to acutely activate the stress response (Samson et al., 2007; Frank et al., 2015, 2019; Hahad et al., 2019; Hayes et al., 2019), which has increasingly been linked to both microglial activity (Frank et al., 2019) and adult hippocampal neurogenesis in recent years (Surget and Belzung, 2021). The significantly higher serum corticosterone observed in noise-exposed mice at 0 HPN (i.e., immediately after NE) compared with the control demonstrates a transient stress response induced by noise in the present study.

A recent study has shown that microglia express a diverse array of receptors, which also allows them to respond to stress hormones derived from peripheral and central sources (Frank et al., 2019). Emerging evidence indicates that the activation of microglia by stress results in profound morphological and functional changes that could disrupt neuronal function, impair neurogenesis, and alter cognitive and emotional behavior (Kreisel et al., 2014; Delpech et al., 2015; Frank et al., 2019; Sanguino-Gomez et al., 2021). Furthermore, studies have demonstrated that there are cortisol receptors throughout the hippocampus (Korz and Frey, 2003) and showed that the proliferation of granule cell precursors, and ultimately the production of new granule cells, is dependent on the levels of circulating adrenal steroids (McEwen, 1999). Stressful experiences, which are known to elevate circulating levels of glucocorticoids, inhibit the proliferation of granule cell precursors (Tanapat et al., 1998; McEwen, 1999; Surget and Belzung, 2021) and the survival of newborn neurons (Thomas et al., 2007). Based on these findings and our data, we speculate that the transient stress response caused by NE in our study may have been associated with mediating the functional response of microglia in each target brain region at the early stage after NE (within 1 week); however, most of the target brain regions showed no abnormal microglia phenotype at 14DPN, suggesting that the transient stress response is insufficient to explain the reappearance of microglia phenotypic abnormalities and aberrant hippocampal neurogenesis in the long term after NE (28DPN) and thus further highlights the major contribution of permanent hearing loss to the prolonged phenotype change of microglia and hippocampal neurogenesis.

In summary, this study observed the effects of acute high-intensity NE on the stress response, hearing threshold, hippocampal neurogenesis, and functional phenotypes of hippocampal and central auditory system microglia. Our results illustrated early but transient and late but progressive microglial activation and hippocampal neurogenesis impairment, an early but transient increase in serum corticosterone, and a permanent moderate-to-severe degree of hearing loss in noise-exposed mice. These findings indicate for the first time that both the stress response and HL are potential contributors to the time-dependent alterations of microglia and hippocampal neurogenesis, which both consist of an early but transient change and a late but enduring change following NE. The noise-induced stress response may be involved with early but transient alterations, while noise-induced permanent HL is more likely to be responsible for late but enduring changes, i.e., microglial dysfunction and hippocampal neurogenesis disturbances that are associated with many neurological diseases (including cognitive impairment and dementia). The microglial overactivation observed in the auditory ROIs may have represented neuronal circuit remodeling closely associated with the acquired peripheral hearing loss caused by high-intensity NE, although it also suggested that the acquired peripheral hearing loss disturbance of auditory input caused by NE induced hippocampal microglia dysfunction and neurogenesis decline. Therefore, we can suggest some reasonable hypotheses based on the available data. Given the pervasiveness of noise in modern life (from commercial, industrial and recreational sources, etc.) and the insidious and progressive nature of neurodegenerative disorders, our observations further highlight that raising awareness of the adverse health impact of noise represents a promising and cost-effective strategy to prevent or delay the onset of dementia.

## Data Availability Statement

The original contributions presented in the study are included in the article/supplementary material, further inquiries can be directed to the corresponding author/s.

## Ethics Statement

The animal study was reviewed and approved by the University Committee for Laboratory Animals of Southeast University, China.

## Author Contributions

LL and GT designed the experiment and supervised the project. CY, DX, RL, CF, and YW managed the mouse cohorts. HL, SS, SZ, and QL conducted the noise exposure and ABR measurements. QL, XY, and CW conducted the sample preparation for the histological study and ELISA. QL, HL, and XY performed the histological procedures and data collection. HZ, HL, and CW conducted the data analysis. QL, HZ, YX, and LL were involved in the data interpretation. QL and LL contributed to the manuscript writing. All authors contributed to the article and approved the submitted version.

## Conflict of Interest

The authors declare that the research was conducted in the absence of any commercial or financial relationships that could be construed as a potential conflict of interest.

## Publisher’s Note

All claims expressed in this article are solely those of the authors and do not necessarily represent those of their affiliated organizations, or those of the publisher, the editors and the reviewers. Any product that may be evaluated in this article, or claim that may be made by its manufacturer, is not guaranteed or endorsed by the publisher.
